# Supplementary material for: Are carbon emissions trading and green financial instruments synergistic? -Comprehensive quantitative research based on content analysis
Source: PLoS One. 2024 Mar 7;19(3):e0298601. doi: 10.1371/journal.pone.0298601 (PMC10919601; doi:10.1371/journal.pone.0298601)
Supplement: S1 Data — (ZIP) [file pone.0298601.s001.zip › Data/地级市Z_F工作报告.zip!/┴╔─■/║∙┬1⁄2╡║╩╨/└┤╫╘╣1⁄2╓┌║┼í╛┬φ┐╦╩2╛▌═°í┐_2019.docx]

各位代表：

　　现在，我代表市人民政府向大会报告工作，请予审议，并请市政协委员和其他列席会议的同志提出意见。

　　一、2018年工作回顾

　　2018年，我们坚持以习近平新时代中国特色社会主义思想为指导，深入贯彻党的十九大和十九届二中、三中全会精神，学习贯彻习近平总书记关于深入推进东北振兴重要讲话精神，坚持稳中求进工作总基调，坚持高质量发展，按照新发展理念和“四个着力”“三个推进”要求，以“重强抓”专项行动为抓手，深入实施“五大区域发展战略”，加快推进“一带五基地”建设，全力打好三大攻坚战，狠抓各项任务落实，保持了经济社会持续健康发展。

　　预计地区生产总值800亿元，增长6.5%;规模以上工业增加值225亿元，增长13%;固定资产投资245亿元，增长10%;一般公共预算收入82.3亿元，增长13%;社会消费品零售总额535亿元，增长7%;城镇居民人均可支配收入29926元，增长7%;农村居民人均可支配收入12548元，增长7%。

　　(一)全力以赴稳增长，不断巩固发展态势

　　稳预期，地区生产总值始终保持6%以上增长，规模以上工业增加值、一般公共预算收入始终保持10%以上增长。稳投资，固定资产投资增幅始终保持全省前列，总量实现位次前移。稳金融，全市金融机构存款余额比年初增长16.3%，贷款余额比年初增长15.4%。稳就业，全市城镇新增就业1.5万人，城镇登记失业率4.8%，低于省控指标。一年来，我们顶住了经济下行压力，保持了稳中有进、稳中向好、稳步提升的良好势头。

　　(二)坚定不移促改革，不断增强经济发展活力

　　完成县乡财政管理体制改革，在全省率先实现乡镇金库全覆盖全运行，夯实了乡镇经济基础，财政收入超年初基数近1倍，消除集体经济“空壳村”294个，中央电视台《新闻联播》报道了我市改革经验。完成事业单位改革，公益性事业单位精简到152家，经营性事业单位转企改制111家。完成市级政府机构改革，支持司法体制改革，政府部门整合到29家。推进“放管服”改革，实现“32证合一”、124项证照即办即给、312项“最多跑一次”，有效解决“回迁难”“办证难”等问题，完成全国营商环境试评价。推进园区管理体制改革，6家园区成立运营公司。推进国资国企改革，成立混合所有制进出口贸易集团，完成驻葫央企“三供一业”分离移交和3户厂办大集体改革。深入推进“三去一降一补”，完成“地条钢”专项整治，彻底关闭30万吨以下小煤矿，商品房去库存240万平方米。一年来，我们克服了各种阻力，啃下了很多“硬骨头”，为振兴发展注入了强劲动力。

　　(三)扬长补短调结构，不断提升产业发展质量

　　第一，调整产业发展方向。优化农业结构，发展设施农业，推进“七彩绥中”“澜盛农业”等重点项目，新增设施农业2万亩;发展农产品深加工，农产品加工企业达到114家。调整工业结构，发展新材料产业，葫芦岛铝业60万吨模板、北方铜业15万吨电解铜等重点项目竣工投产，一个新的“百亿”产业已经形成;发展军民融合产业，成立军民融合产业园，建立产业发展联盟，认定首批军民融合企业28户，实施海洋核动力平台、AP1000核管道等18个重点项目，构建了军民融合创新发展体系;发展泳装产业，开工建设泳装生产基地、运营基地，培育龙头企业，举办国际泳装展，发布国际泳装指数。发展高端服务业，引进了和润德医养干细胞、日式温泉小镇等一批高档康养类项目，集聚了一批商贸餐饮、休闲度假、旅游会展等新兴产业，邴家湾服务业新业态集聚区快速崛起、蓬勃发展。壮大旅游产业，欢乐龙湾、加勒比水乐园等重点项目投入运营，成功举办辽宁省海岛旅游节、关东民俗冰雪节，打造了一批标志性旅游新产品，旅游吸引力、影响力明显提升。

　　第二，调整区域合作方向。对接京津冀，承接产业转移，引进北京航数智能材料、北宇智能制造等项目31个，总投资117亿元。加强与江苏对口合作，推进航锦科技、高邮化学添加剂等3个项目，总投资10.2亿元，与常州建立互派干部挂职交流制度。推进区域协作，加入辽宁沿海经济带六城市协同发展行动计划，与京津冀城市共同成立旅游联盟。强化招才引智，设立人才发展基金1亿元，新建院士专家工作站14个，引进博士后7名、外国专家12名，人才工作取得明显成果。

　　第三，调整对外开放方向。加强与日韩经贸合作，洽谈推进日本JBC日式民宿、韩国西岸株式会社医疗康养等17个项目，其中8个项目签约落地;面向德国等欧洲国家招商，引进德国西门子管线压缩机、德钾盐二期等项目，打开了日韩和欧洲招商新局面。开拓“一带一路”国家市场，在俄罗斯建设矿业产业园，与文莱、泰国、斯里兰卡等国家开展海外项目合作，对中东欧国家出口增长8.9倍。一年来，我们优化了产业结构，提升了经济外向度，为打造“特色发展新高地”奠定了坚实基础。

　　(四)尽心竭力惠民生，不断提升民生保障水平

　　第一，全力推进脱贫攻坚。落实“五个一批”，实施“五个联动”，改造危房3858户，实现3.9万人稳定脱贫、65个村销号。兴城碱厂乡荣获全国脱贫攻坚组织创新奖，兴城产业扶贫、南票危房改造经验在全省推广。

　　第二，坚决整治环境污染。深刻吸取东戴河虚假整改教训，狠抓环保问题整改，中央环保督察33项整改任务中，青山水库环保工程、县(市)区污水处理厂提标改造等15项已完成。办结中央环保督察“回头看”信访案件393件，办结率99.5%。国家海洋督察、“绿盾”专项行动、水源地专项督查问题整改均已完成序时进度。

　　第三，切实解决民生问题。深入实施“三城同盛”，推进同城同质，六大工程全部完工，老城面貌持续改善，新城品质不断提升，连山、龙港、兴城实现三位一体、互联互通、深度融合。积极回应民生关切，改造棚户区3643户，实施“暖房子”工程36.6万平方米，改造老城区管网8公里，建成高标准社区服务站6个;开通外环路公交，更新新能源公交车185台，建设停车位800个，22项民生工程如期完成。全力解决历史难题，海航路、海翔路、海韵路3条干线全部竣工通车，打通了新老城区快速干道，拉开了城市发展格局。

　　第四，着力完善保障体系。坚持民生优先，提高养老金、城乡低保标准，民生支出占一般公共预算支出77%。推进义务教育均衡发展，改造农村地区薄弱学校47所，义务教育阶段“超大班额”全部消除。完善基层文化设施，建成基层综合性文化服务中心427个。推进公立医院改革，建立县域医联体7个，实现基层医疗机构全覆盖。一年来，我们坚持以人民为中心的发展思想，切实解决了一批群众关心、社会关注的热点难点问题。

　　(五)积极主动防风险，不断改善振兴发展环境

　　第一，有效管控重大风险。全力化解政府性债务，累计化债186亿元，全市综合债务率持续降低。开展金融领域专项整治行动，有效防控了企业债务风险、金融风险。克服财政支出压力，安排财政预算，强化措施保障，确保养老金始终按时足额发放，做到了“不伸手、不穿底、不拖欠”。

　　第二，着力加强社会治理。持续开展生产安全专项整治，事故起数、死亡人数分别下降29.3% 和12.9%。扎实推进“扫黑除恶”专项斗争，打掉黑恶势力团伙15个。深入开展信访减存控增三年攻坚行动，化减积案425件，化解进度位居全省前列。严密防控非洲猪瘟疫情，启动猪肉政府储备，有效稳定市场，确保安全供应。持续加强食品药品安全监管，建立警务云平台、猎鹰巡逻队等立体化治安防控体系。一年来，我们防范了各类风险隐患，维护了社会和谐稳定，人民群众安全感、满意度不断提升。

　　此外，我们狠抓中央三次巡视和省委巡视反馈问题整改，政府系统整改任务全部按时间节点完成。认真办理人大代表建议、政协提案，按时办复率100%，满意和基本满意率99.8%。积极推进“涉军停偿”工作，66个“停偿”项目全部完成。妥善处置建昌“11·22”重大刑事案件、湖南非法运砂船翻沉、虹螺山森林火灾等突发事件。成功举办国际沙滩排球邀请赛、兴城海峡马拉松等大型赛事，承办省第九届少数民族传统体育运动会。

　　各位代表，成绩来之不易，凝聚着全市人民的智慧和汗水。在此，我代表市政府，向全市各族人民，向全体人大代表、政协委员，向各民主党派、工商联、无党派人士、人民团体，向驻葫部队指战员、武警官兵、公安干警、中省直单位和各界朋友，致以崇高的敬意和衷心的感谢!

　　对标高质量发展要求，我市振兴发展还存在一些明显短板。一是经济总量小，产业结构不协调，新兴产业规模小，民营经济发展不充分。二是重点领域改革不够深入，改革力度不均衡、效果不明显。三是开放合作领域不宽、层次不高，港口能级低，经济外向度差，沿海优势未得到有效发挥。四是个别部门和干部不担当、不作为问题突出，办事难现象仍然存在，营商环境仍需进一步改善。对此，我们将采取有效措施，着力加以解决。

　　二、2019年工作安排

　　2019年是新中国成立70周年、建市30周年，是决胜全面建成小康社会的关键之年。我们要深入贯彻习近平总书记系列重要讲话精神，认真落实中央、省委经济工作会议和市委五届十一次全会要求，以全新的思想、务实的举措、更高的追求，挖掘潜能、转换动能、积蓄势能，全力推动经济社会高质量发展。

　　今年政府工作的指导思想是：高举习近平新时代中国特色社会主义思想伟大旗帜，全面贯彻党的十九大和十九届二中、三中全会精神，深入落实习近平总书记关于深入推进东北振兴重要讲话精神，统筹推进“五位一体”总体布局，协调推进“四个全面”战略布局，坚持稳中求进工作总基调，坚持新发展理念，坚持高质量发展，以供给侧结构性改革为主线，深入实施“五大区域发展战略”，加快推进“一带五基地”建设，继续打好三大攻坚战，抓好稳增长、促改革、调结构、惠民生、防风险各项工作，努力实现“四大发展目标”，奋力开创葫芦岛全面振兴、全方位振兴发展新局面!

　　今年政府工作的总体目标是：地区生产总值增长6.8%，规模以上工业增加值增长8%，固定资产投资增长10%，一般公共预算收入增长10%，社会消费品零售总额增长7%，城镇和农村居民人均可支配收入增长7%。

　　我们将着力做好以下九个方面工作：

　　(一)深入学习习近平总书记重要讲话精神，努力践行新理念新思想新战略

　　深入学习贯彻习近平总书记关于深入推进东北振兴重要讲话精神，牢固树立“四个意识”，坚定“四个自信”，坚决做到“两个维护”。开展“解放思想推动高质量发展大讨论”，聚焦“四个短板”“六项重点工作”，围绕省市“1+8+N”文件精神，抓好实践融合，狠抓任务落实，把习近平总书记重要讲话精神贯彻落实到政府各项工作中。

　　(二)深入开展“重强抓”专项行动，确保完成各项任务目标

　　坚持以“重强抓”专项行动统揽政府工作，把工作任务全部纳入专项行动中，实现政府工作系统化、目标化、标准化、台账化。健全完善“交办单、督办单、催办单”三单制度，严格执行“评价、问责”两个办法，强化考核结果运用，形成鲜明工作导向。

　　(三)把握结构调整方向，扎实推进经济高质量发展

　　第一，壮大工业，夯实高质量发展根基。发展优势产业，延长产业链条，建立新兴产业体系，打造特色发展新高地。一是重点发展以铝型材为代表的新材料产业，葫芦岛铝业全线投产，完成产值160亿元，开工建设年产45万吨车用零部件项目，加快新能源汽车整车骨架项目落地。二是重点发展以涉核装备为代表的军民融合产业，开工建设海洋核动力平台、伊菲科技复合陶瓷装甲等重点项目，推进精谷科技与中船重工715所科研成果转化合作，争做军民融合发展先行者。三是重点发展以中盈游艇为代表的旅游装备产业，加快推进旅游装备产业园项目。四是重点发展以泵阀为代表的流体仪表制造产业，推进华电森源智能电网设备、新锦机涡轮机械等项目建设。五是重点发展以核电为代表的新能源产业，加快推进徐大堡核电一期、兴城抽水蓄能电站等重点项目。六是重点发展以粮食加工为代表的农产品深加工产业，开工建设30万吨燃料乙醇、35万吨大豆深加工等项目。七是重点发展以泳装为代表的运动服饰产业，扶持斯达威等龙头企业，培育范德安等知名品牌，引进华歌尔等国外企业，努力成为泳装标准制定者和发展风向标。

　　第二，优化服务业，提升高质量发展水平。扩大消费市场，释放消费潜力，形成新的经济增长点。建设邴家湾服务业新业态集聚区，加快推进日式民宿、俊安泉海御龙湾等13个重点项目。发展高端服务业，成立矿产品交易中心，引进希尔顿等高端酒店，新增品牌连锁店5家、社区便利店20家、限上企业60家。发展康养产业，重点建设和润德医养干细胞、正业健康产业园等优质项目。升级商贸流通业，开工建设东城传化物流公路港项目，兴宫中街投入运营。发展文化创意产业，做大会展经济，培育文化创意产业基地。壮大旅游产业，开工建设北京嘉艺影视基地、宏跃水上乐园等24个重点项目，推进与江西三清山集团旅游项目合作，规划建设东戴河休闲度假区、九门口和锥子山长城自助游营区等五大旅游集聚区，旅游总收入增长20%以上。

　　第三，做精农业，补足高质量发展短板。保证粮食安全，发展高效农业，新建高效精品科技示范果园50个，发展优质花生117万亩、优质杂粮29万亩。发展设施农业，建设市级设施蔬菜示范园3个，食用菌、蓝莓等高效农业园20个，新增设施农业2万亩。发展农产品加工业，重点抓好福康花生、菊花女蔬菜等5个重点项目。发展品牌农业，发挥地理标志产品优势，打造葫芦岛苹果、兴城花生、绥中白梨、建昌核桃、南票大枣五大特色品牌。完善物流配送体系，发展“互联网+农业”、电子商务、仓储物流等新产业新业态，促进农业提质增效。

　　第四，扶持民营经济，增强高质量发展动力。深入贯彻习近平总书记在民营企业座谈会上的重要讲话精神，全力以赴帮助民营企业解决实际困难、加快转型升级。支持民营企业发展壮大，实现“个转企”600户、“小升规”30户、“规升巨”2户。落实民营经济发展政策，推进电力直接交易，降低企业交易成本，新增融资110亿元，市县政府采购支出30%面向民营企业。放宽准入领域，营造公平环境，鼓励民营资本参与国资国企改革、重大项目建设。搭建服务企业平台，完善项目代办机制，加强诚信体系建设，构建亲清政商关系，促进民营经济加快成长。

　　第五，实施项目牵动，筑牢高质量发展基础。实施亿元以上重点项目220个，总投资2232亿元。其中，以农产品深加工为重点的农业项目8个;以转型升级为方向的工业项目56个;以新业态新模式为主体的服务业项目24个;以水上游乐、休闲康养为代表的旅游业项目9个;能源交通、基础设施等其他项目123个。建立项目管家全覆盖制度，加强土地、资金、人才等要素保障，强化领导包扶、督查考核，确保葫芦岛有色20万吨电解锌、锦西石化25亿元炼油结构调整等46个重点项目开工建设，大唐热电联产、七星建筑材料等71个项目竣工投产。

　　(四)紧扣改革创新主题，着力提升振兴发展活力

　　第一，持续改善营商环境。加强硬件建设，改善政务服务环境，完成市行政服务中心异地改建，市本级所有审批部门、审批职能全部进驻。提升服务质量，推进行政审批“三集中三到位”改革，70%以上政务服务事项实现“一窗受理”，建成全市一体化网上政务服务平台，网上办理率90%以上。完成工程建设项目审批制度改革，审批时限压缩至90个工作日以内。全面复制推广辽宁自贸区经验，为振兴发展创造一流营商环境。

　　第二，深化重点领域改革。完成县乡政府机构改革，理顺部门职能，优化机构设置，保障政府系统高效运转。深化供给侧结构性改革，持续推进“三去一降一补”。深化国资国企改革，完成7户厂办大集体改革、3户“僵尸企业”处置、5户市属企业集团子公司混合所有制改革。深化财税、金融、土地、市场准入、社会管理等专项改革。

　　第三，加大科技创新力度。继续实施高新技术企业三年倍增计划，研发经费占比达到1.1%，高新技术产品增加值增长15%以上，科技成果转化35项以上。加强高新区建设，培育创新型企业，新增高新技术企业5家，建成产业技术创新平台2个。实施“百千万”重点人才工程，开展博士后挂职服务、“万名专家葫芦岛行”等活动，为振兴发展提供人才保障。

　　(五)拓展对外开放空间，打造服务“一带一路”示范区

　　第一，对接“一带一路”。融入中东欧“16+1”建设，加强与沿线国家经贸技术合作，加快建设俄罗斯矿业产业园，重点推进芳纶科技柬埔寨耐高温材料、厚能科技泰国新能源电池等海外合作项目。把握与招商局集团合作机遇，争取绥中港纳入全省港口资源整合。发展东戴河临港产业，推进港产城一体化。提升葫芦岛港港口能级，完善海关特殊监管区，提高对外开放水平。

　　第二，强化对外经贸合作。深耕日韩等国家市场，重点面向日韩中小企业招商，加快日本正丸酿造食品加工、高丽精密株式会社地铁配件等10个项目签约落地，建设韩国中小企业园。强化与德国经贸合作，重点围绕装备制造产业招商，推进西门子管线压缩机、技术服务中心等重点项目。深化区域协作，加强与常州对口合作，推进与沿海城市协同发展。承接京津冀产业转移，引进项目30个以上，建设对接京津冀桥头堡。全年利用外资、引进内资增长15%以上。

　　第三，扩大进出口贸易。应对中美经贸摩擦，依托外贸转型升级基地、泳装海外营销网络和保利集团等军贸企业，带动国华能源石化设备、力天重工军工装备等新产品出口，开拓中东欧、中东、非洲等新兴市场。发挥市进出口贸易集团载体作用，扩大世界优质商品进口，打造区域性进口商品集散地。

　　第四，加快沿海经济带建设。落实中央和省市各项政策，以产业园区为依托，深化公司化改革，加强招商引资，加快柯漫热敏新材料、虹京碳酸锂等重点项目建设，努力把沿海经济带建设成为支撑振兴发展的新引擎。

　　(六)对标中央决策部署，坚决打好三大攻坚战

　　第一，打赢精准脱贫攻坚战。攻克难点，坚决完成3.2万贫困人口脱贫、17个贫困村销号，建昌县实现脱贫摘帽。发展产业，加快推进泳装下乡、扶贫车间等重点扶贫开发项目，保障贫困人口收入，实现稳定脱贫。实施精准脱贫，开展精准识别“回头看”，确保实现真脱贫、不返贫。

　　第二，全力抓好污染防治。实施治水工程，深入落实河长制，加强全域水质监测，确保河流监测断面水质全部达标，完成女儿河上游钼超标治理示范工程。实施蓝天工程，逐步拆除建成区内10~20吨燃煤锅炉，开展柴油货车超标排放等专项整治，确保PM2.5浓度和空气优良天数达标。实施土壤防治工程，治理航锦科技、稻池村、八家子土地污染，整治闭坑矿山、尾矿库。狠抓中央环保督察和“回头看”、国家海洋督察、“绿盾”专项行动和水源地专项督查等问题整改，坚决完成各项目标任务。

　　第三，防范化解重大风险。强化财政预算管理，细化预算编制，硬化预算约束，有效增加资金供给。安排财政预算，处置有效资产，弥补养老金缺口，坚决防控养老金支付风险，确保按时足额发放。严禁新增隐性债务，妥善化解存量债务，完成化债任务40亿元，坚决防范政府债务风险。严厉打击非法集资犯罪活动，防范金融市场异常波动，确保不发生区域性、系统性金融风险。

　　(七)补齐乡村振兴短板，发展壮大县域经济

　　第一，县域推进园区建设。坚持“三区共建”，扶持中小企业，加快项目建设，扩大县域经济总量。壮大工业产业园，实施全钢重卡子午线轮胎、川铁电气产业基地等千万元以上工业项目35个。发展农产品加工园区，实施海源水产品加工、德国精酿啤酒等百万元以上农业产业化项目20个。培育服务业集聚区，重点建设绥中商贸集聚区、绥中物流产业园、建昌特色商业街、兴城服务业新业态产业带。

　　第二，乡镇突出“飞地经济”。落实实施意见，明确发展目标，确定主导产业，推动差异化、特色化发展。建设标准化厂房，市本级建设10 万平方米，各县(市)区不少于5万平方米。加强招商引资，新增“飞地”项目100个以上，全年完成固定资产投资14亿元以上，每个乡镇至少引进1个项目。强化政策支持，市级财政每年安排1亿元专项资金，金融机构每年投入融资额度20亿元，扶持“飞地经济”发展。

　　第三，村屯壮大集体经济。培育经济组织，建立合作社、行业协会200个，新增农事企业50家，带动村级集体经济发展。加大扶持力度，用好农村改革资金、乡村振兴项目资金，发展村级集体经济试点15个，扶持农业经营主体38个。发展多种经营，通过土地流转、股份合作，发展“种养加”产业、设施农业，增加村级集体收入，全年消除“空壳村”150个。

　　第四，加强县城环境整治。完善城区道路交通、地下管网等基础设施，提升城市承载力。开展环境综合治理，集中整治露天市场、城乡结合部，改造老旧小区，加强社区建设，提高县城精细化管理水平。

　　第五，完善农村基础设施。加强交通路网建设，维修改造干线公路100公里，新建改造农村公路500公里，新建农村候车亭200个。加强农村环境综合整治，推进“厕所革命”，完成农村改厕10698座。完善公共服务设施，改善农村人居环境，发展一批特色乡村、特色小镇。

　　(八)践行为民服务宗旨，全力保障和改善民生

　　第一，坚定实施“三城同盛”。突出生态优先、规划引领、陆海统筹，实施三大专项规划。加快海绵城市建设，完成雨污分流改造8公里。实施“三河”及入海口综合治理工程，消除城区黑臭水体，扩建老区污水处理厂。开工建设垃圾焚烧发电厂，推进固体废弃物处理、生活垃圾清储运一体化，提高人居环境质量。完善城市基础设施，实施小街小巷修复工程，新建改造供水供暖管网7公里。加强城市管理，提升城市品位，争创全国文明城市、国家卫生城市。

　　第二，提升社会保障能力。落实就业政策，推动高校毕业生、退役军人、就业困难群体稳定就业，实现建档立卡贫困家庭大学毕业生全部就业，新增城镇就业1.4万人，城镇登记失业率控制在5.2%以内。实施全民参保登记，推动社保精准扩面，提高城乡低保、特困供养标准。提升住房保障能力，实施棚户区改造364户，发放公租房租赁补贴1910户，贫困户D级危房全部完成改造。

　　第三，完善公共服务体系。加快义务教育城乡一体化进程，推进县管校聘教师管理体制改革，消除义务教育阶段“大班额”159个，一乡至少建设一所公办中心幼儿园。推进“健康葫芦岛”建设，建成标准化乡镇卫生院22个、标准化村卫生室100个。拓展文化惠民工程，完善基层文化设施，加强文物保护工作，举办第五届全民运动会。

　　第四，创造平安和谐环境。树立安全理念，落实安全责任，狠抓生产安全和公共安全，坚决遏止重特大安全事故发生。加强基层民主政治建设，强化社会治安综合治理，持续开展“扫黑除恶”专项斗争，建设平安葫芦岛。提升防灾减灾救灾和应急处置能力，狠抓信访矛盾减存控增，确保社会大局和谐稳定。

　　(九)坚持党的全面领导，坚决抓好政府自身建设

　　第一，坚定政治立场，忠诚干事。切实履行全面从严治党主体责任，主动落实“一岗双责”，全面贯彻中央八项规定精神，严格执行廉洁自律准则和纪律处分条例，深入推进政府系统党风廉政、意识形态建设和反腐败斗争，营造风清气正的政治生态。

　　第二，主动接受监督，规矩干事。坚持市委统一领导，坚决贯彻市委决策部署，严格执行市人大及其常委会决议决定，主动接受市人大依法监督和工作监督，积极支持市政协履行职能，自觉接受市政协民主监督。坚持依法治市、依法行政，严格执行“三重一大”集体决策制度，提升政府工作科学化、民主化、法治化水平。

　　第三，努力提高本领，务实干事。牢固树立担当意识，弘扬实干精神，增强“八个本领”，完善绩效考核体系，强化政府规则刚性约束，坚决整肃庸政懒政怠政行为，切实提高政府执行力，树立政府公信力。

　　各位代表!葫芦岛全面振兴、全方位振兴走进了新时代，踏上了新征程，让我们紧密团结在以习近平同志为核心的党中央周围，不忘初心、牢记使命，以新气象新担当新作为，奋力开创工作新局面，为决胜全面建成小康社会而努力奋斗!
